# Housemaid’s Knee (Prepatellar Septic Bursitis)

**DOI:** 10.7759/cureus.10398

**Published:** 2020-09-11

**Authors:** Masaya Sato, Takashi Watari

**Affiliations:** 1 Department of Orthopaedic Surgery, Unnan City Hospital, Shimane, JPN; 2 Department of Internal Medicine, Shimane University Hospital, Izumo, JPN

**Keywords:** prepatellar septic bursitis, housemaid’s knee, lymphangitis

## Abstract

An 83-year-old Japanese tatami craftsman with underlying diabetes mellites who complained of severe pain and feeling of warmth in his right knee, with mild chills. Fluid accumulation was seen in his prepatellar bursa and *Staphylococcus aureus* was detected in his synovial fluid culture, confirming the diagnosis of prepatellar septic bursitis. Prepatellar bursitis is well known as housemaid's knee, which is caused by inflammation of the prepatellar bursa among people who spend long periods of time kneeling such as housemaids, clergy, and gardeners.

## Introduction

When evaluating a patient with knee swelling, a physician must consider whether the swelling is attributed to mono- or polyarticular pathology; this is also essential to the differential diagnosis of intra-articular or extra-articular soft tissue infection [1]. Prepatellar bursitis, also known as housemaid's knee, is caused by inflammation of the prepatellar bursa in individuals who spend long periods kneeling, such as housemaids, clergy, and gardeners [2]. However, this infection often presents with mono-arthritis-like findings and requires careful palpation and confirmation by intra- and extra-articular punctures. This article presents the case of a patient with delayed diagnosis of housemaid’s knee and discusses how the delay in diagnosis could have been prevented.

## Case presentation

**Figure 1:**
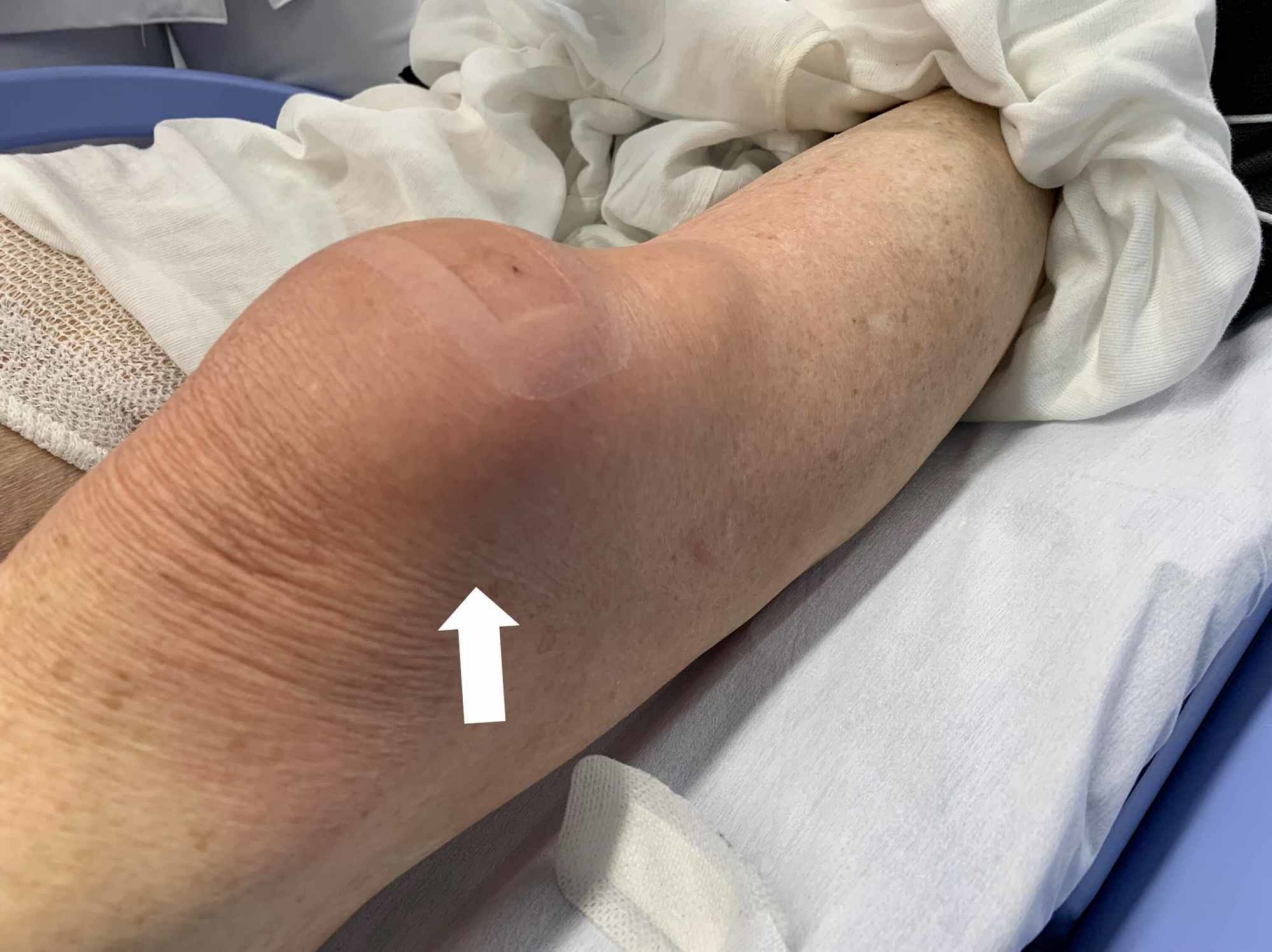
Inflammation of the front right knee

**Figure 2:**
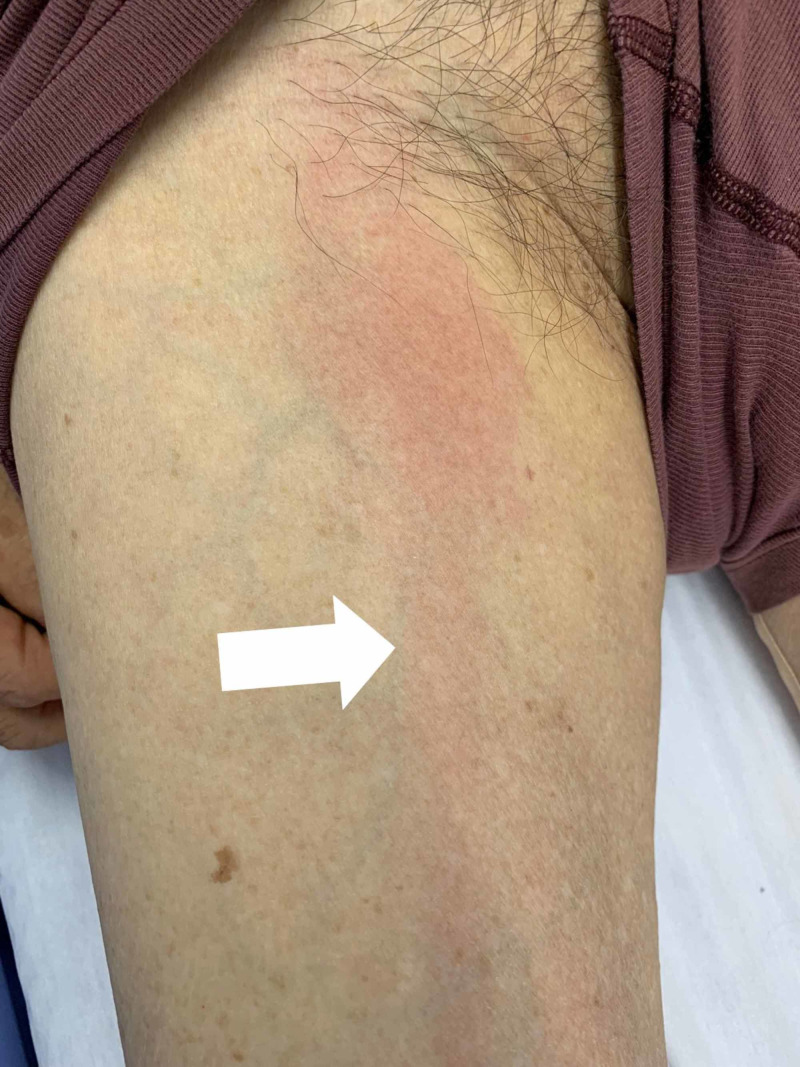
Redness extending from the medial thigh to the groin.

**Figure 3:**
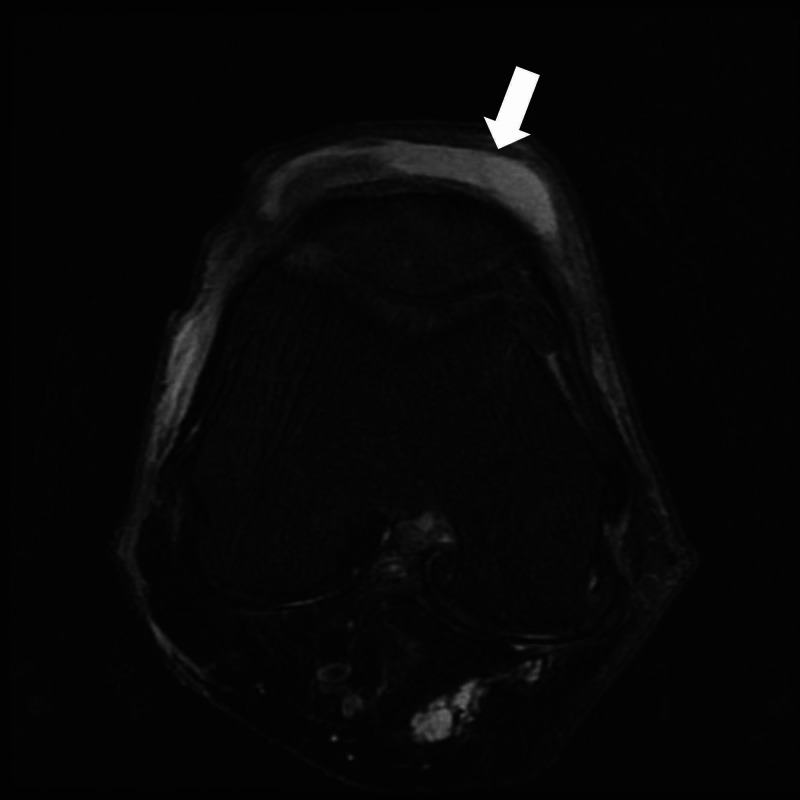
T2 fat suppression MRI scan (Axial view) showed hyperintensity area in the prepatellar bursa of right knee.

An 83-year-old male Japanese tatami craftsman with a history of diabetes mellitus presented to our emergency room with complaints of severe pain and warmth in the right knee, accompanied by mild chills [3]. Three weeks prior, he had noticed a tiny piece of wood lodged in his right knee. Marked swelling, tenderness, and redness were observed at the knee's anterior aspect during the initial visit. The patient was diagnosed with pseudogout and discharged; knee aspiration was not performed. The patient presented to the emergency room the following day complaining of severe pain. In addition to inflammation in the front right knee, limited range of motion (Figure 1) and redness extending from the right buttock to the medial thigh (Figure 2) were observed, indicating lymphangitis. Knee magnetic resonance imaging showed no significant abnormalities in the joint space (Figure 3), although fluid accumulation was observed in the prepatellar bursa.

Fluid aspiration was performed, *Staphylococcus aureus* was detected in the synovial fluid culture, and the patient was hospitalized. First-generation cephem antibiotics were intravenously administered, leading to full recovery without sequelae.

## Discussion

Herein, we report the case of an elderly patient whose diagnosis of housemaid’s knee was delayed and who fully recovered after hospitalization and intravenous antibiotic administration. The main symptoms typically include pain, peribursal erythema, and warmth. Fever only occurs in 40-44% of cases [4,5]. Gram staining must be performed to obtain the correct diagnosis, and the aspirated bursal synovial fluid must be cultured if septic bursitis is suspected, as it is difficult to differentiate between an infectious and a non-infectious disease without laboratory test results [6]. *S. aureus* is the primary causative organism in more than 80% of septic bursitis cases confirmed based on bacterial culture findings [3,7]. For severe infections or in an immunocompromised host, intravenous antibiotic treatment should be considered [7]. A skin abrasion can cause acute lymphangitis with infection at a distal site, such as lower-leg cellulitis. This finding may be accompanied by lymphadenitis, characterized by redness and tender streaks extending proximally. By considering the lymphatic flow anatomically, we can determine whether the infection site is positioned more distally [8,9]. We initially considered diseases such as pseudogout, which has a high epidemiological frequency but is relatively uncommon outside the knee joint. We believe that the delay in this case’s diagnosis was due to availability of information bias during diagnosis [10], confirmation bias, which ignored the findings of suspected lymphangitis [11], and failure to perform arthrocentesis and Gram staining of the fluid within the knee.

## Conclusions

The clinically relevant message pertaining to this case is that when a patient who could be a candidate for housemaid’s knee presents with knee swelling, aspiration and culture are advisable to ascertain the characteristics of the fluid and arrive at the appropriate diagnosis. However, many physicians often diagnose such patients with pseudogout because they fail to perform said test. We believe that this is a fairly common pitfall when encountering mono-arthritis and fever symptoms in elderly patients.
